# Other PHAC publications

**DOI:** 10.24095/hpcdp.45.3.04

**Published:** 2025-03

**Researchers from the Public Health Agency of Canada also contribute to work published in other journals and books. Look for the following articles published in 2024 and 2025:**

Chaput JP, Morin CM, Robillard R, Carney CE, Dang-Vu T, Davidson JR, […] **Lang JJ**. Trends in nighttime insomnia symptoms in Canada from 2007 to 2021. Sleep Med. 2025;125:21-6. https://doi.org/10.1016/j.sleep.2024.11.025

**Chen R, Gilbert NL**, Dub . Adult influenza vaccination coverage before, during and after the COVID-19 pandemic in Canada. BMC Public Health. 2024;24(1):3357. https://doi.org/10.1186/s12889-024-20854-6

Clayborne ZM, **Wong SL, Roberts KC, Prince SA, Garipy G**, Goldfield GS, […] **Lang JJ**. Associations between social media use and positive mental health among adolescents: findings from the Canadian Health Behaviour in School-aged Children Study. J Psychiatr Res. 2025;181:333-9. https://doi.org/10.1016/j.jpsychires.2024.11.071

**Doan N, Lang JJ, Roberts KC**, Manyanga T, Rainham DG, **Capaldi CA, Butler G, Prince SA, Srugo SA**. Investigating the independent and synergistic associations between neighbourhood greenness and physical activity in relation to perceived mental health among adults in Canada. Int J Environ Health Res. 2024:1-12. https://doi.org/10.1080/09603123.2024.2426712

Jibb L, **Laverty M**, Johnston DL, Rayar M, Truong TH, Kulkarni K, […] **Kaur J, Winch N**, et al. Association between socioeconomic factors and childhood acute lymphoblastic leukemia treatment- and survival-related outcomes in Canada. Pediatr Blood Cancer. 2025; 72(2):e31472. https://doi.org/10.1002/pbc.31472

**Kamal A**, Balachandra T. A review of accidental residential fire death investigations in Alberta 2012–2021. Can Soc Forensic Sci J. 2024:1-15. https://doi.org/10.1080/00085030.2024.2430422

**Liu L, Contreras G, Pollock NJ, Thompson W**. Suicidal ideation among Canadian adults during the third year of the COVID-19 pandemic compared to pre- and early pandemic periods. Ment Health Prev. 2024;36:200379. https://doi.org/10.1016/j.mhp.2024.200379

**Liu L, Pollock NJ, Contreras G, Xu Y, Thompson W**. Self-harm hospitalizations and neighbourhood level material and social deprivation in Canada: an ecological study. BMC Psychiatry. 2024;24(1):859. https://doi.org/10.1186/s12888-024-06316-8

Macneil A, Taunque A, Leo SN, Li G, **de Groh M, Jiang Y**, et al. The mental health toll of the COVID-19 pandemic on older adults with migraine: a prospective analysis of depression using the Canadian Longitudinal Study on Aging. J Pain Res. 2024;17:3845-66. https://doi.org/10.2147/JPR.S469798

**Richmond N**, Ornstein A, **Tonmyr L, Dzakpasu S, Nelson C, Pollock NJ**. Child maltreatment mortality in Canada: an analysis of coroner and medical examiner data. Child Abuse Negl. 2025;159:107127. https://doi.org/10.1016/j.chiabu.2024.107127

Roy E, Jaeger B, Evans AM, Turetsky KM, O’Shea BA, Petersen MB, […] **Gretton JD**, et al. A contest study to reduce attractiveness-based discrimination in social judgment. J Pers Soc Psychol. 2024. https://doi.org/10.1037/pspa0000414

Rushton B, Lemieux CJ, Scott DJ, Halpenny EA, Tompkins J, Jones B, […] **Prince SA**, et al. Future-proofing nature-based tourism in Canada: a horizon scan of emerging challenges. Curr Issues Tourism. 2024. https://doi.org/10.1080/13683500.2024.2431526

**Slota JA, Lamoureux L, Frost KL, Sajesh BV, Booth SA**. Single-cell transcriptomics unveils molecular signatures of neuronal vulnerability in a mouse model of prion disease that overlap with Alzheimer’s disease. Nat Commun. 2024;15(1):10174. https://doi.org/10.1038/s41467-024-54579-2

